# Genuine Multipartite Entanglement in the 3-Photon Decay of Positronium

**DOI:** 10.1038/s41598-017-15356-y

**Published:** 2017-11-10

**Authors:** Beatrix C. Hiesmayr, Pawel Moskal

**Affiliations:** 1 Faculty of Physics, University of Vienna, Boltzmanngasse 5, 1090 Vienna, Austria; 2 Institute of Physics, Jagiellonian University, Cracow, Poland

## Abstract

The electron-positron annihilation into two photons is a standard technology in medicine to observe e.g. metabolic processes in human bodies. A new tomograph will provide the possibility to observe not only direct *e* ^+^ *e* ^−^ annihilations but also the 3 photons from the decay of ortho-positronium atoms formed in the body. We show in this contribution that the three-photon state with respect to polarisation degrees of freedom depends on the angles between the photons and exhibits various specific entanglement features. In particular genuine multipartite entanglement, a type of entanglement involving all degrees of freedom, is subsistent if the positronium was in a definite spin eigenstate. Remarkably, when all spin eigenstates are mixed equally, entanglement –and even stronger genuine multipartite entanglement– survives. Due to a “*symmetrization*” process, however, *Dicke*-type or W-type entanglement remains whereas *GHZ*-type entanglement vanishes. The survival of particular entanglement properties in the mixing scenario may make it possible to extract quantum information in the form of distinct entanglement features, e.g., from metabolic processes in human bodies.

## Introduction

The detection of the two high energetic photons resulting from the annihilation of an electron and a positron is a well-established successful technology to image metabolic processes in living bodies (PET: Positron Emission Tomography). PET application is used in many different fields of medicine, e.g. oncology, cardiology, radiation therapy or neurology. In recent years, PET instrumentation has undergone a steady multifaceted evolution and the improvements include new hardware, new reconstruction methods and implementation of time-of-flight techniques^1–7^. Without doubt PET serves as an important tool in imaging metabolic processes based on the sensitivity to tracers (positron-emitting radionuclides) injected into the body or tissue.

Electron-positron annihilations may occur either directly or via the creation of positronium atoms (a bound state of an electron and positron). Positronium^8–11^ can be in an anti-symmetric spin state (para-positronium) or a symmetric spin state (ortho-positronium). Charge conjugation implies that in the first case it decays into an even number of photons (2*γ*, 4*γ*, …) and in the other case into an odd number of photons (3*γ*, 5*γ*, …). Due to kinematics and smallness of the fine-structure constant the 2*γ* and 3*γ* cases are the two most likely options. During routine PET imaging, positronium atoms are formed copiously inside the human body and therefore 3*γ*-decays occur also frequently. Even in water the production of ortho-positronium has a probability equal to about 25%^12^ and is expected to be more than 38% in tissue^13^. Three-photon events, however, have never been used in tomography because of technical limitations of standard PET devices. A new prototype, called J-PET (Jagiellonian-PET)^14–19^, has been shown to meet all technical requirements in performing such a measurement for the first time.

This paper investigates the entanglement in the polarisation degrees of freedom of the three photons resulting from the decay of the ortho-positronium. Both for a fixed spin quantization direction of the positronium as well as the case of equal mixing. Photons are fascinating quantum systems, having spin one, but due to their mass-less property there is a nontrivial coupling between the spin and momentum properties. The most appropriate single-photon description remains controversial. A recent framework describing all single-photon states and single-photon observables by POVMs (positive-operator valued measurements) can be found in ref.^20^. In this contribution we restrict ourselves to the polarisation degrees of freedom and are interested in the correlation of three photons with energies that ranges from 0 to 511 keV. Entanglement and in particular multipartite entanglement is a highly investigated field that has the potential to become a new technology. This paper makes a step towards investigating what type of entanglement is present in the three-photon state generated by the decay of ortho-positronium. This may one day result in obtaining not only local information, namely *where* in a tissue the positronium decayed, but also revealing quantum information which may serve as a new quantum marker for specific biological processes.

Note that entanglement seems to play an important role in biological systems as e.g. observed in the light harvesting complexes, e.g. ref.^21^, in bird navigation (European robin)^22,23^ or in olfaction^24^. Let us emphasize here that these works have led to a paradigm change in the sense that life may be too “warm and wet” for quantum phenomena to endure.

The paper is organized as follows. First we introduce the 3-photon state resulting from the decay of ortho-positronium. Then we analyse the multipartite entanglement of the pure state scenario including a discussion of the distribution of entanglement among the three photons. Then we proceed to the mixed scenario proving that entanglement is not lost. This is followed by a summary and outlook.

## The states relevant in ortho-positronium decays

Ortho-positronium decays mainly into 3 photons. The resulting state of the 3 photons depends on the quantization direction $\hat{\overrightarrow{n}}$ of the ortho-positronium’s spin state (total spin 1). If the third component ${s}_{\hat{\overrightarrow{n}}}$ is zero, the state is given by

1 $$|{{\rm{\Psi }}}_{{s}_{\hat{\overrightarrow{n}}}=0}\rangle =\frac{1}{\sqrt{N}}\,\mathop{\underbrace{(\cos ({{\rm{\Phi }}}_{plane}{)1}^{\otimes 3}+\,\sin ({{\rm{\Phi }}}_{plane}){{\rm{\sigma }}}_{x}^{\otimes 3})]}}\limits_{{\rm{phas}}\,{\rm{edependence}}}\cdot \,\mathop{\underbrace{{\hat{ {\mathcal R} }}_{pol}({\tilde{{\rm{\Theta }}}}_{ab},\,{\tilde{{\rm{\Theta }}}}_{bc})}}\limits_{{\rm{polarisation}}\,{\rm{operator}}}\,\,\mathop{\underbrace{|{\rm{\Psi }}{\rangle }_{abc}}}\limits_{\mathrm{GHZ}-\mathrm{state}}$$

with the normalisation

2 $$\begin{array}{c}N=\frac{1}{2}\,(9+\,\cos \,2{\tilde{{\rm{\Theta }}}}_{ab}+\,\cos \,\mathrm{(2}{\tilde{{\rm{\Theta }}}}_{ab}+2{\tilde{{\rm{\Theta }}}}_{bc})+\,\cos \,2{\tilde{{\rm{\Theta }}}}_{bc}\\ \quad \,\,\,\,\,-4(\cos \,{\tilde{{\rm{\Theta }}}}_{ab}+\,\cos ({\tilde{{\rm{\Theta }}}}_{ab}+{\tilde{{\rm{\Theta }}}}_{bc})+\,\cos \,{\tilde{{\rm{\Theta }}}}_{bc}))\end{array}$$

and the polarisation operator

3 $$\begin{array}{c}{\hat{ {\mathcal R} }}_{pol}({\tilde{{\rm{\Theta }}}}_{ab},\,{\tilde{{\rm{\Theta }}}}_{bc})=\sum _{i,j,k=0}^{1}\,({(-1)}^{k}{\sin }^{2}\frac{{\tilde{{\rm{\Theta }}}}_{ab}}{2}+{(-1)}^{j}{\sin }^{2}(\frac{{\tilde{{\rm{\Theta }}}}_{ab}}{2}+\frac{{\tilde{{\rm{\Theta }}}}_{bc}}{2})+{(-1)}^{i}{\sin }^{2}\frac{{\tilde{{\rm{\Theta }}}}_{bc}}{2})\\ \quad \quad \quad \quad \quad \quad \,\,\,\times \,|ijk{\rangle }_{abc}\langle ijk{|}_{abc}\end{array}$$

and the state

4 $$\begin{array}{rcl}|{\rm{\Psi }}{\rangle }_{abc} & = & \mathrm{|000}{\rangle }_{abc}-\mathrm{|110}{\rangle }_{abc}-\mathrm{|011}{\rangle }_{abc}-\mathrm{|101}{\rangle }_{abc}\\  & = & |{\varphi }^{-}{\rangle }_{ab}\otimes \mathrm{|0}{\rangle }_{c}-|{\psi }^{+}{\rangle }_{ab}\otimes \mathrm{|1}{\rangle }_{c}\\  & = & \mathrm{|0}{\rangle }_{a}\otimes |{\varphi }^{-}{\rangle }_{bc}-\mathrm{|1}{\rangle }_{a}\otimes |{\psi }^{+}{\rangle }_{bc}\\  & = & |{\varphi }^{-}{\rangle }_{ac}\otimes \mathrm{|0}{\rangle }_{b}-|{\psi }^{+}{\rangle }_{ac}\otimes \mathrm{|1}{\rangle }_{b}\\  & = & |+++{\rangle }_{abc}+|---{\rangle }_{abc}\,,\end{array}$$

written in the computational basis {|0〉, |1〉} which is defined as the eigenstates with respect to the internal frame of each photon and may be identified with the linear polarised states |*H*〉, |*V*〉. The states |*ϕ* ^±^〉 = |00〉 ± |11〉, |*ψ* ^±^〉 = |01〉 ± |10〉 are the Bell states (not normalized). The states |+/−〉 correspond to the right-/left-handed circular polarised photons with respect to the choice of internal space of each photon $|+/-\rangle =\frac{1}{\sqrt{2}}\{|0\rangle \pm i|1\rangle \}$. Assuming that a particular photon *i* travels in the *z*-direction, then |0〉, |1〉 can be identified also with the electric field components in the *x*,*y*-direction, respectively. This state |Ψ〉_*abc*_ is also known to be a Greenberger-Horn-Zeilinger (GHZ) state (discussed later).

**Figure 1:**
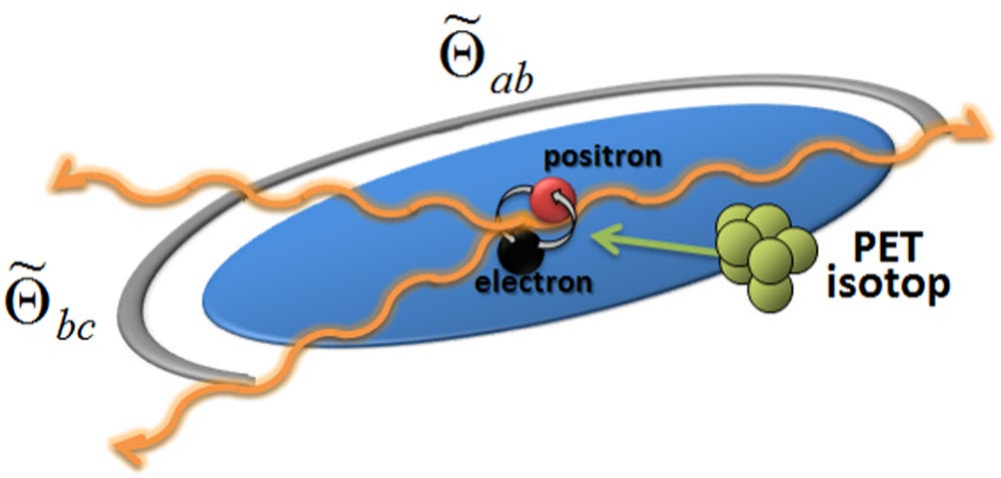
This graphic shows schematically how from an isotope typically used in standard PET-therapy, e.g. FDG-18 (fludeoxyglucose), positronium is generated that decays into three photons with wave vectors which lie in one plane due to energy and momentum conservation.

Due to momentum and energy conservation the three momenta of the photons have to lie in a plane, which will be denoted as the *decay plane*. The operator ${\hat{ {\mathcal R} }}_{pol}({\tilde{{\rm{\Theta }}}}_{ab},\,{\tilde{{\rm{\Theta }}}}_{bc})$ covers the symmetries superposed by the decay process on the polarisation degrees of freedom, where ${\tilde{{\rm{\Theta }}}}_{ij}$ corresponds to the angles between photon *i* and *j*, that all lie in the decay plane (see Fig. 1). Further restrictions due to momentum and energy conservation on these angles are discussed at the end of this section.

The phase ${{\rm{\Phi }}}_{plane}\in [0,\,\frac{\pi }{2})$ is the angle between the spin-quantization direction $\hat{\overrightarrow{n}}$ of the ortho-positronium and the decay plane formed by the three photons’ momenta. Therefore, the other two possible eigenstates of ortho-positronium ${s}_{\hat{\overrightarrow{n}}}=\pm 1$ are obtained by three local rotations, i.e.

5 $$\begin{array}{rcl}|{{\rm{\Psi }}}_{{s}_{\hat{\overrightarrow{n}}}=+1}\rangle  & = & {{\rm{\sigma }}}_{x}^{\otimes 3}\,|{{\rm{\Psi }}}_{{s}_{\hat{\overrightarrow{n}}}=0}\rangle \\  & = & \frac{1}{\sqrt{N}}(\sin ({{\rm{\Phi }}}_{plane}{\mathrm{)1}}^{\otimes 3}+\,\cos ({{\rm{\Phi }}}_{plane}){{\rm{\sigma }}}_{x}^{\otimes 3})\cdot {\hat{ {\mathcal R} }}_{pol}({\tilde{{\rm{\Theta }}}}_{ab},\,{\tilde{{\rm{\Theta }}}}_{bc})\,|{\rm{\Psi }}{\rangle }_{abc},\\ |{{\rm{\Psi }}}_{{s}_{\hat{\overrightarrow{n}}}=-1}\rangle  & = & {{\rm{\sigma }}}_{y}^{\otimes 3}\,|{{\rm{\Psi }}}_{{s}_{\hat{\overrightarrow{n}}}=0}\rangle \\  & = & \frac{1}{\sqrt{N}}(\sin ({{\rm{\Phi }}}_{plane}){{\rm{\sigma }}}_{z}^{\otimes 3}+\,\cos ({{\rm{\Phi }}}_{plane}){{\rm{\sigma }}}_{y}^{\otimes 3})\cdot {\hat{ {\mathcal R} }}_{pol}({\tilde{{\rm{\Theta }}}}_{ab},\,{\tilde{{\rm{\Theta }}}}_{bc})\,|{\rm{\Psi }}{\rangle }_{abc}.\end{array}$$

A detailed computation of the state $|{{\rm{\Psi }}}_{{s}_{\hat{\overrightarrow{n}}}}\rangle$ based on quantum electrodynamic (QED) for the case Φ_*plane*_ = 0 can be found in ref.^25^.

Momentum and energy conservation constrains also the allowed values of the angles ${\tilde{{\rm{\Theta }}}}_{ab},{\tilde{{\rm{\Theta }}}}_{bc}$ of the three momenta forming the decay plane. Energy conservation in the rest mass system of the positronium leads to ($\hslash c\equiv 1$)

6 $${\omega }_{a}+{\omega }_{b}+{\omega }_{c}=E,$$

which fixes one energy value of the three photons. Momentum conservation relates the values of energy of any two photons *a*, *b* to its solid angle by

7 $$\cos \,{\tilde{{\rm{\Theta }}}}_{ab}=\frac{\frac{1}{2}-\frac{{\omega }_{a}}{E}-\frac{{\omega }_{b}}{E}+\frac{{\omega }_{a}{\omega }_{b}}{{E}^{2}}}{\frac{{\omega }_{a}{\omega }_{b}}{{E}^{2}}}.$$

**Figure 2:**
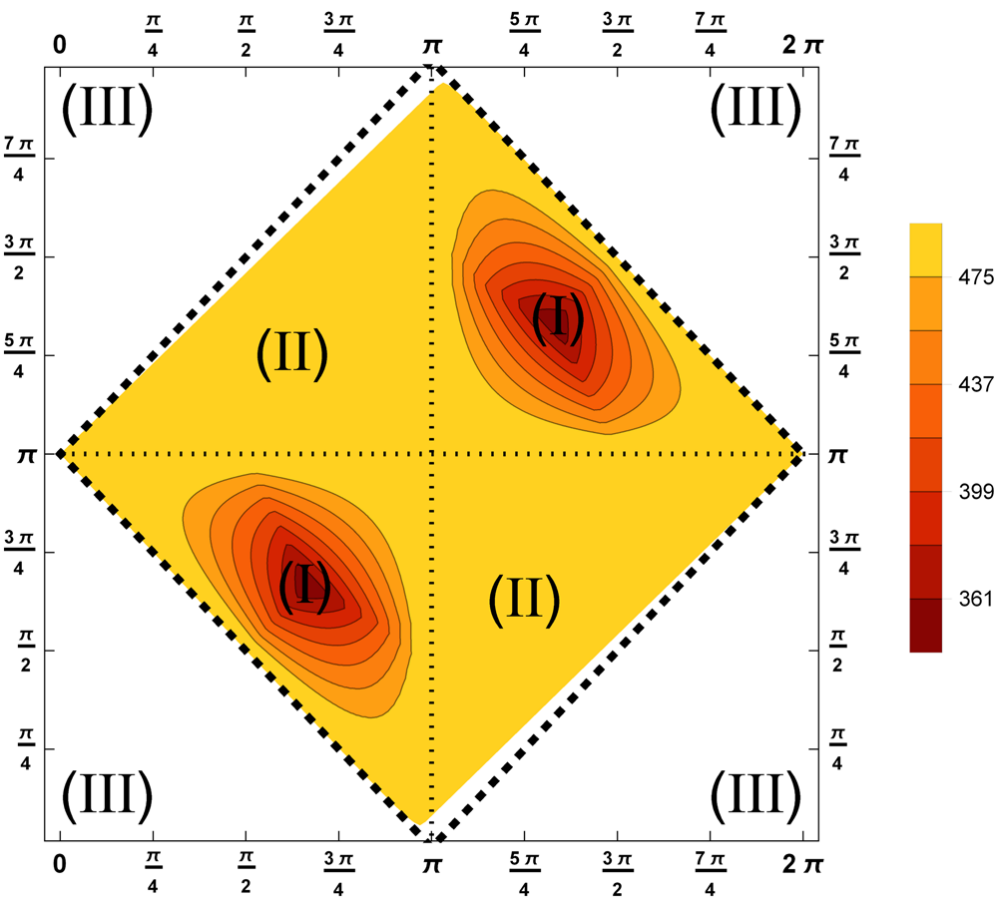
These contour plots show the maximum taken over all single photon energies of three photons for the allowed angles ${\tilde{{\rm{\Theta }}}}_{ab}$ (*x*-axis) and ${\tilde{{\rm{\Theta }}}}_{bc}$ (*y*-axis). Three kinematically different regions emerge. A forbidden region (III) where the momentum conservation does not hold since all wave vectors point into one half of the plane. Another forbidden region (II) where one photon has zero energy. And a physically relevant region (I), here the energies are not extremal. This plot agrees with Figure 8 of ref.^18^, where also a Dalitz plot is shown for this case.

A solution is only obtained if the function on the right-hand side is in the interval [−1, 1]. The lower bound −1 implies that a single photon can have at most half of the total energy *E*, and the upper bound +1 bounds the sum of both energies to half of the total energy *E*. The possible range of angles is shown in Fig. 2. The kinematics thus singles out the region denoted by (*I*). In summary, the full parameter space is not physically attainable due to energy and momentum conservation.

## Results

### Entanglement properties of the pure 3-photon-states

In the following we want to consider the polarisation degrees of freedom of the three-photon state, independent of the kinematical constraints. From this perspective the states $|{{\rm{\Psi }}}_{{s}_{\hat{\overrightarrow{n}}}}\rangle$ are tripartite qubit systems (qubit... two-level systems).

Any entanglement of a tripartite state can be classified according to the *k*-separability^26^ (for a more recent overview of the subtleties concerning the classification of multipartite states see e.g. ref.^27^). If a pure *n*-partite state can be written in the form

8 $$|\psi \rangle =|{\varphi }_{1}\rangle \otimes |{\varphi }_{2}\rangle \otimes \ldots \otimes |{\varphi }_{k}\rangle $$

with *k* ≤ *n*, it is called for *k* = *n fully separable*, for 1 < *k* < *n partially separable* (*k-separable*) or for *k* = 1 *fully entangled* (1*-separable*). There is a straightforward extension to mixed states, i.e., if a mixed state can be written as a convex combination of at least *k*-separable states, i.e. (*p*_*i*_ ≥ 0)

9 $$\rho =\sum _{i}\,{p}_{i}\,{\rho }_{i}^{1}\otimes {\rho }_{i}^{2}\otimes \ldots \otimes {\rho }_{i}^{k}$$

with *k* ≤ *n*, it is classified as in the case for pure states. Note that 1-separable states are also called *genuinely multipartite entangled* states and these states are the most interesting ones with respect to outperforming algorithms exploiting classical physics.

This classification is certainly not fine enough. Already for the simplest case, three qubits, we have two physically very different subclasses of 1-separable states or genuinely multipartite entangled states: The GHZ-states (GHZ... Greenberger, Horne, Zeilinger)

10 $$|GHZ\rangle =\frac{1}{\sqrt{2}}(\mathrm{|000}\rangle +\mathrm{|111}\rangle )$$

and the Dicke states (*W* states called in the case of three qubits), e.g.,

11 $$|W\rangle =\frac{1}{\sqrt{3}}(\mathrm{|001}\rangle +\mathrm{|010}\rangle +\mathrm{|100}\rangle )\mathrm{.}$$

Both are obviously a particular generalization of the maximally entangled Bell states, but their physical properties are very different. For example, if one subsystem is traced out, the entanglement is fully lost in the case of *GHZ*-type entanglement, in contrast to the *W*-type entanglement, where the subsystems are entangled. It has been shown that the *GHZ*-type entanglement can be utilized for multipartite quantum cryptography^28–30^ whereas for Dicke-type entanglement no such schemes have been found that outperform bipartite entangled systems. Dicke-type entanglement is often present in condensed matter systems^31^ or is produced by a double down conversion process resulting in four genuinely multipartite entangled photons^32,33^. For single neutrons in an interferometric setup, three degrees of freedom can be engineered, i.e. spin, path and energy, for which both types of genuine multipartite entanglement have been generated experimentally^34^. Recently, also atoms in a solid have been proven to be genuinely multipartite entangled^35,36^. Topological and nematic phase transitions in spin chains are shown to be ruled by genuine multipartite entanglement^37–39^, even if bipartite entanglement dies out^40^.

Our first aim is to analyze the entanglement of the three photons resulting from the ortho-positronium decay. Without loss of generality we can set Φ_*plane*_ = 0 and ${s}_{\hat{\overrightarrow{n}}}=0$ since the entanglement properties do not depend on local unitaries (if not mixed). Thus the state under investigation depends only on the two azimuth angles

12 $$|{\psi }_{pure}({\tilde{{\rm{\Theta }}}}_{ab},{\tilde{{\rm{\Theta }}}}_{bc})\rangle ={\hat{ {\mathcal R} }}_{pol}({\tilde{{\rm{\Theta }}}}_{ab},\,{\tilde{{\rm{\Theta }}}}_{bc})\,|{\rm{\Psi }}{\rangle }_{abc}.$$

For pure states, well-established bipartite entanglement criteria serve the purpose of revealing all the entanglement. For mixed states, since entanglement detection is a NP-hard problem^41^, only necessary but not sufficient criteria can be found. The HMGH-framework^42^ provides such necessary criteria to classify the different types of multipartite entanglement. This framework connects local observables, density matrix elements, to distinct types of entanglement and is therefore also experimentally feasible.

In ref.^42^ it was proven that the following criterion holds for all *k*-separable states *ρ*

13 $${Q}_{k}(\rho )=\sqrt{\langle \chi |{\rho }^{\otimes 2}{P}_{total}|\chi \rangle }-\sum _{\{\alpha \}}\,{(\prod _{i=1}^{k}\langle \chi |{P}_{{\alpha }_{i}}^{\dagger }{\rho }^{\otimes 2}{P}_{{\alpha }_{i}}|\chi \rangle )}^{\frac{1}{2k}}\le 0,$$

where |*χ*〉 = |*χ*_1_〉⊗|*χ*_2_〉 is an arbitrary fully separable state, ${P}_{{\alpha }_{i}}$ is a permutation operator permuting the *α*_*i*_-th elements of |*χ*_1_〉 and |*χ*_2_〉 and the sum runs over all *k*-partitions {*α*}. And the total permutation acts as *P*_*total*_|*χ*_1_〉⊗|*χ*_2_〉 = |*χ*_2_〉⊗|*χ*_1_〉. Obviously, if this inequality is violated the state *ρ* cannot be *k*-separable. Note that the reverse argument does not hold since a non-violation does not necessarily imply *k*-separability. Consequently, these criteria are necessary but not sufficient criteria for *k*-separability. Since the above criteria obviously depend on the choice of the fully separable state |*χ*〉 and the chosen basis of *ρ*, one always has to optimize over local unitaries in order to obtain the optimum (which is performed in all the following computations).

For three qubits a value of *Q*_*k*=3_(*ρ*) greater than zero detects entanglement of *ρ* and a value of *Q*_*k*=2_(*ρ*) greater than zero detects the state to be genuinely multipartite entangled. It also turns out that *Q*_*k*=2_(*ρ*) is the one that gives the highest value for the *GHZ*-state, i.e. *Q*_*k*=2_(|*GHZ*〉) = 1. In strong contrast to the *W* states which give *Q*_*k*=2_(|*W*〉) = 0.629. In the following we therefore denote this criterion by *Q*_*GHZ*_ := *Q*_*k*=2_. And by *Q*_*SEP*_ := *Q*_*k*=3_ the criterion detecting entanglement but not necessarily genuine multipartite entanglement. Explicitly, we can rewrite the criteria which detects entanglement if the value is greater than zero by

14 $$\begin{array}{c}{Q}_{SEP}(\rho )=2\cdot |\langle \mathrm{000|}\rho \mathrm{|111}\rangle |-2(\langle \mathrm{001|}\rho \mathrm{|001}\rangle \langle \mathrm{010|}\rho \mathrm{|010}\rangle \langle \mathrm{011|}\rho \mathrm{|011}\rangle \\ \quad \quad \quad \quad {\langle \mathrm{100|}\rho \mathrm{|100}\rangle \langle \mathrm{101|}\rho \mathrm{|101}\rangle \langle \mathrm{110|}\rho \mathrm{|110}\rangle )}^{\frac{1}{6}}\,\mathrm{.}\end{array}$$

The criterion, which detects genuine multipartite entanglement if the value is greater than zero and is maximized for any *GHZ*-state, can be rewritten as

15 $$\begin{array}{c}{Q}_{GHZ}(\rho )=2\cdot (|\langle \mathrm{000|}\rho \mathrm{|111}\rangle |-\sqrt{\langle \mathrm{110|}\rho \mathrm{|110}\rangle \langle \mathrm{001|}\rho \mathrm{|001}\rangle }\\ \quad \quad \quad \quad \,-\sqrt{\langle \mathrm{101|}\rho \mathrm{|101}\rangle \langle \mathrm{010|}\rho \mathrm{|010}\rangle }-\sqrt{\langle \mathrm{011|}\rho \mathrm{|011}\rangle \langle \mathrm{100|}\rho \mathrm{|100}\rangle })\,\mathrm{.}\end{array}$$

This formulation reveals the very working of the criteria, i.e. that the only off-diagonal elements of these criteria are exactly the only non-zero off-diagonal element of the *GHZ*-state, and the negative terms are the diagonal elements of *ρ* which are all zero in the case of the *GHZ*-state.

A criterion to optimize for the *W*-type entanglement can also be derived via the HMGH-framework^42^. The same strategy as above can be used, i.e. choosing the non-zero elements of the *W*-state, i.e. |001〉, |010〉 or |100〉 for the fully separable state |*χ*_1_〉 and |*χ*_2_〉. Since we have now three combinations we can add these three inequalities to have a symmetric criterion. In ref.^43^, however, it was shown that one can obtain a stricter inequality if one adds a further constraint coming from the positivity condition. For 3-particles with two degrees of freedoms the following criterion detects genuine multipartite entanglement if greater than zero and attains its maximal value for the *W*-state

16 $$\begin{array}{rcl}{Q}_{W}(\rho ) & = & \mathrm{2|}\langle \mathrm{001|}\rho \mathrm{|010}\rangle |+\mathrm{2|}\langle \mathrm{001|}\rho \mathrm{|100}\rangle |+\mathrm{2|}\langle \mathrm{010|}\rho \mathrm{|100}\rangle |-(\langle \mathrm{001|}\rho \mathrm{|001}\rangle \\  &  & +\langle \mathrm{010|}\rho \mathrm{|010}\rangle +\langle \mathrm{100|}\rho \mathrm{|100}\rangle +2\sqrt{\langle \mathrm{000|}\rho \mathrm{|000}\rangle \cdot \langle \mathrm{011|}\rho \mathrm{|011}\rangle }\\  &  & +2\sqrt{\langle \mathrm{000|}\rho \mathrm{|000}\rangle \cdot \langle \mathrm{101|}\rho \mathrm{|101}\rangle }+2\sqrt{\langle \mathrm{000|}\rho \mathrm{|000}\rangle \cdot \langle \mathrm{110|}\rho \mathrm{|110}\rangle })\,\mathrm{.}\end{array}$$

The positive terms are the only non-zero off-diagonal terms of the *W*-state whereas the negative terms are the only diagonal terms that are zero in the case of the *W*-state. Therefore, this criterion gives the maximum value for the *W*-state. This is in strong contrast to *GHZ*-states which obtain the value ${Q}_{W}(|GHZ\rangle )=\frac{3}{4}$. The separability criterion for both genuinely multipartite entangled states is *Q*_*SEP*_(|*GHZ*〉) = 1 and *Q*_*SEP*_(|*W*〉) = 0.62. A summary–including the positronium case–can be found in Table 1.

**Table 1:** The optimized values of the three entanglement criteria for different three qubit states.

3 Qubits	*Q*_*GHZ*_	*Q*_*W*_	*Q*_*SEP*_
|*GHZ*〉	1	$$\frac{3}{4}$$	1
|*W*〉	0.628	1	$$\frac{2}{3}$$
$ma{x}_{{\tilde{{\rm{\Theta }}}}_{ab},{\tilde{\Theta }}_{bc}}|{\psi }_{pure}\rangle$	0.76	0.83	0.89
	$({\tilde{{\rm{\Theta }}}}_{ab}=\frac{15\pi }{8},\,{\tilde{{\rm{\Theta }}}}_{bc}=\frac{\pi }{4})$	$({\tilde{{\rm{\Theta }}}}_{ab}=\frac{15\pi }{8},\,{\tilde{{\rm{\Theta }}}}_{bc}=\frac{\pi }{4})$	$({\tilde{{\rm{\Theta }}}}_{ab}=\frac{\pi }{16},\,{\tilde{{\rm{\Theta }}}}_{bc}=\frac{\pi }{16})$
$|{\psi }_{pure}(\frac{2\pi }{3},\frac{2\pi }{3})\rangle$	0.58	0.67	0.67
${\rho }_{mixed}(\frac{1}{3},\,0)(\frac{2\pi }{3},\,\frac{2\pi }{3})$	0	0.5	0.17

Obviously these criteria are measurable by local observables since they depend only on density matrix elements, for example

17 $$\begin{array}{rcl}\langle \mathrm{000|}\rho \mathrm{|111}\rangle  & = & {\langle {{\rm{\sigma }}}_{x}\otimes {{\rm{\sigma }}}_{x}\otimes {{\rm{\sigma }}}_{x}\rangle }_{\rho }-{\langle {{\rm{\sigma }}}_{x}\otimes {{\rm{\sigma }}}_{y}\otimes {{\rm{\sigma }}}_{y}\rangle }_{\rho }-{\langle {{\rm{\sigma }}}_{y}\otimes {{\rm{\sigma }}}_{x}\otimes {{\rm{\sigma }}}_{y}\rangle }_{\rho }\\  &  & -{\langle {{\rm{\sigma }}}_{y}\otimes {{\rm{\sigma }}}_{y}\otimes {{\rm{\sigma }}}_{x}\rangle }_{\rho }-i({\langle {{\rm{\sigma }}}_{x}\otimes {{\rm{\sigma }}}_{x}\otimes {{\rm{\sigma }}}_{y}\rangle }_{\rho }+{\langle {{\rm{\sigma }}}_{x}\otimes {{\rm{\sigma }}}_{y}\otimes {{\rm{\sigma }}}_{x}\rangle }_{\rho }\\  &  & +{\langle {{\rm{\sigma }}}_{y}\otimes {{\rm{\sigma }}}_{x}\otimes {{\rm{\sigma }}}_{x}\rangle }_{\rho }-{\langle {{\rm{\sigma }}}_{y}\otimes {{\rm{\sigma }}}_{y}\otimes {{\rm{\sigma }}}_{y}\rangle }_{\rho }),\end{array}$$

where *σ*_*i*_ are the Pauli matrices. This makes the criteria very experimenter friendly since they are attainable by local measurements only and do not need state tomography.

**Figure 3:**
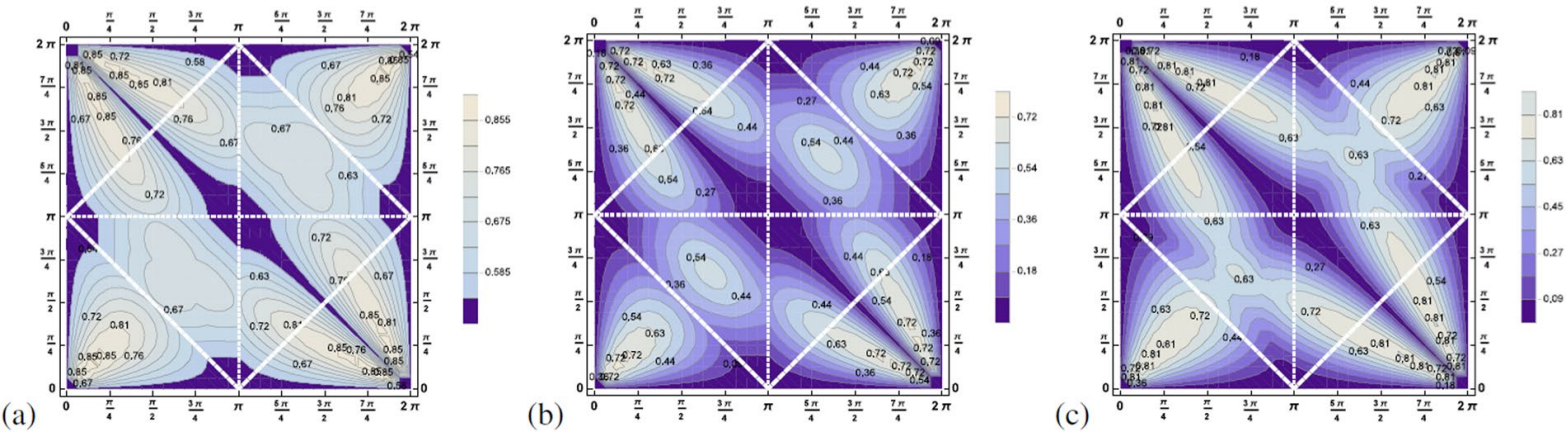
These contour plots show the function (**a**) *Q*_*SEP*_, (**b**) *Q*_*GHZ*_ and (**c**) *Q*_*W*_ for the pure state $|{\psi }_{pure}({\tilde{{\rm{\Theta }}}}_{ab},\,{\tilde{{\rm{\Theta }}}}_{bc})\rangle$ for each ${\tilde{{\rm{\Theta }}}}_{ab}$ (*x*-axis) and ${\tilde{{\rm{\Theta }}}}_{bc}$ (*y*-axis) (optimized via local unitaries). *Q*_*SEP*_ is always greater than zero, indeed even $\ge \frac{1}{2}$, thus proving entanglement for all possible decay scenarios. The quantities detecting genuine multipartite entanglement *Q*_*W*_, *Q*_*GHZ*_ are greater than zero, thus detecting genuine multipartite entanglement, however, their values differ.

Deriving *Q*_*SEP*_ for the three-photon state we find ${Q}_{SEP}(|{\psi }_{pure}({\tilde{{\rm{\Theta }}}}_{ab},{\tilde{{\rm{\Theta }}}}_{bc})\rangle )\ge \frac{1}{2}$, i.e. a positive value for all possible angles. Thus proving that the three-photon states resulting from the decay of positronium are always entangled. The maximum is equal to 0.89 and is reached for ${\tilde{{\rm{\Theta }}}}_{ab}=\frac{\pi }{16},\,{\tilde{{\rm{\Theta }}}}_{bc}=\frac{\pi }{16}$ in the non-physical area. The contour plot in Fig. 3(a) shows the details. Whereas the three-photon state is entangled for all possible decay configurations $\{{\tilde{{\rm{\Theta }}}}_{ab},\,{\tilde{{\rm{\Theta }}}}_{bc}\}$, this surprisingly holds true also for genuine multipartite entanglement detected by *Q*_*GHZ*_ or by *Q*_*W*_. Both criteria differ only in the amount of the violation of the inequality, see Fig. 3(b) and (c).

Summarizing, neglecting the kinematical constraints onto the polarisation degrees of freedom, described mathematically by ${R}_{pol}({\tilde{{\rm{\Theta }}}}_{ab},\,{\tilde{{\rm{\Theta }}}}_{bc})$, the resulting three-photon state in the decay process of the positronium would be a pure *GHZ*-state, a maximal entangled three-qubit state. The superimposed constraints result in a dependence of entanglement on the decay angles obeying the indistinguishability of the individual photons.

In the next section we analyse the distribution of the bipartite entanglement between the individual photons, which will answer how these kinematical constraints onto the polarisation degrees of freedom concentrates different types of genuine multipartite entanglement.

### Entanglement of the reduced system

Now we want to investigate how the entanglement is distributed among the individual photons *a*, *b* and *c*. From equation (4) it is obvious that without the operator ${\hat{ {\mathcal R} }}_{pol}({\tilde{{\rm{\Theta }}}}_{ab},\,{\tilde{{\rm{\Theta }}}}_{bc})$ the reduced state is an equal mixture of two Bell states which is a separable state. The kinematic operator weights not the two Bell states separately but each contribution individually. From this the invariance under permutation of the photons is lost, i.e. the three reduced states *ρ*_*ab*_, *ρ*_*ac*_, *ρ*_*bc*_ differ as well as the entanglement content measured by concurrence. Concurrence is an analytically computable entanglement measure for qubit-qubit entanglement. For pure states it simplifies to $C(|\psi \rangle )=\sqrt{\mathrm{2(1}-Tr({\rho }_{i}))}$ where *ρ*_*i*_ is the partial trace of |*ψ*〉 with respect to the subsystem *i*. For a mixed state concurrence is defined by the convex roof, i.e.

18 $$C(\rho )=\mathop{{\rm{\min }}}\limits_{{p}_{i},|{\psi }_{i}\rangle }\{\sum \,{p}_{i}C({\psi }_{i})\,|\,\sum \,{p}_{i}\,|{\psi }_{i}\rangle \langle {\psi }_{i}\,|=\rho \,\,{\rm{with}}\,{p}_{i}\ge 0\}.$$

For bipartite qubits it has been shown that the convex sum equals the value obtained by computing the eigenvalues of $\sqrt{Tr(\rho {{\rm{\sigma }}}_{2}\otimes {{\rm{\sigma }}}_{2}{\rho }^{\ast }{{\rm{\sigma }}}_{2}\otimes {{\rm{\sigma }}}_{2})}$ and taking the maximal eigenvalue minus the remaining ones.

Since our total three-qubit system is a pure state we can directly answer the question of how much bipartite entanglement photon *a* shares with photon *b* and photon *c*. Obviously, if photons *b* and *c* are in a maximal entangled state they have to be separable from photon *a*. Therefore, the entanglement *a* can share with *bc* limits the entanglement of *bc*. This can be quantified with the help of the famous Coffman-Kondu-Wootters tangle *τ*_*a*|*bc*_^44^, i.e.

19 $$C{({\rho }_{ab})}^{2}+C{({\rho }_{ac})}^{2}\le {\tau }_{a|bc}({\psi }_{abc})\,:=4\,{\rm{\det }}\,{\rho }_{a}.$$

For the *GHZ*-state the reduced states are separable states thus the concurrence is zero, whereas the tangle is maximal. The difference between the right-hand side and the left-hand side is maximal. In the case of *W*-states the reduced states have a value of $C=\frac{2}{3}$ and the tangle equals $\tau =\frac{8}{9}$, i.e. the difference is zero. Thus entanglement is distributed also among the subsystems in the case of a *W*-state, whereas for *GHZ*-states no entanglement can be found in the subsystems. Thus the tangle minus the two concurrences quantifies the difference between genuine multipartite entanglement of the *GHZ*-type entanglement and the *W*-type entanglement.

Let us note here, however, another subtle point of multipartite entanglement. Obviously, if a GHZ-state as given in equation (4) is considered, a measurement of one photon in the circular polarized basis ({| + 〉, | − 〉}) leads to a separable state for the two remaining photons, i.e. |++〉 or |−−〉. If the photon is instead measured in the linear polarized basis ({|0〉, |1〉}) the remaining two photons have the result “0” in the Bell state |*ϕ* ^−^〉 or the outcome “1” in the Bell state |*ψ* ^+^〉, i.e. clearly maximally entangled. This perfect correlation between the polarization state of one photon and the entangled state of the two photons implies, under the Einstein-Podolsky-Rosen premises of realism and no action at a distance, that the entangled state of the two photons must represent an element of reality. Whereas the individual photons, which have no well-defined properties, do not correspond to such elements. For a realist this is a surprising feature. In the first scenario the two photons contain individually an element of reality, which is more satisfactory for a realist. Thus by the specific kind of measurement, projecting on linearly or circularly polarized photons, the properties of the two photons and their reality content is switched between entanglement and separability. This can also be understood from the fact that a particular factorisation per se is not favoured over another one; no partition has ontologically a superior status over any other one, therefore there is perfect democracy. However, a measurement or a physical process makes a choice.

Thus let us come back to the 3-photon decay resulting from ortho-positronium. Though the individual concurrences for a given setup $({\tilde{{\rm{\Theta }}}}_{ab},\,{\tilde{{\rm{\Theta }}}}_{bc})$ differ, the difference of the tangle minus the two squared concurrences, *τ*_*i*|*jk*_ − *C*(*ρ*_*ij*_)^2^ − *C*(*ρ*_*ij*_)^2^, has to be equal for all possible permutations of the three photons. This allows the following interpretation: The kinematics of the decay process are such that the individual bipartite entanglement can vary, however, the total amount of entanglement is not allowed to depend on these individual choices. Choosing a larger or smaller amount of bipartite entanglement of a particular pair affects the amount of entanglement of the other two bipartitions obeying a conservation of the total amount of entanglement.

**Figure 4:**
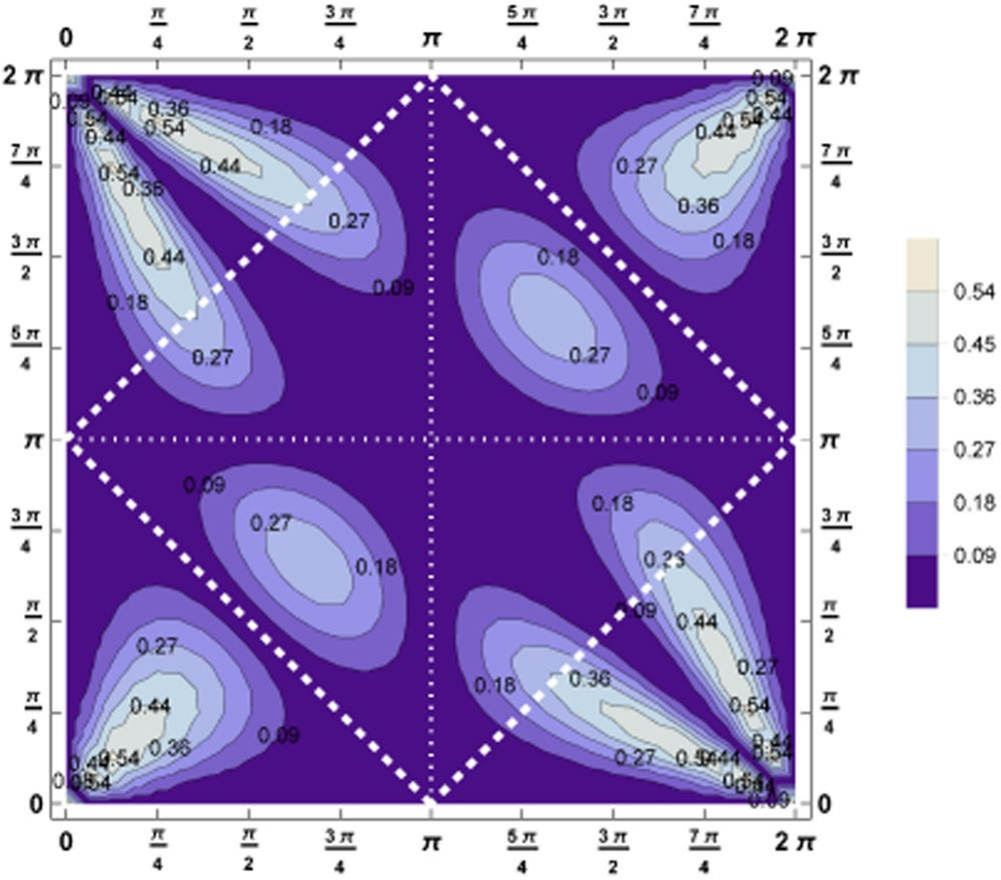
This contour plot shows the tangle minus the two concurrences, *τ*_*i*|*jk*_ − *C*(*ρ*_*ij*_)^2^ − *C*(*ρ*_*ij*_)^2^, which is equal for any permutation of the three photons.

The largest difference between the individual bipartite entanglement and the one shared with both remaining photons overlaps with the regions for which genuine multipartite entanglement maximizes, showing that the *W*-type entanglement is more resistant against the specific setup (angles) respecting the indistinguishability of the photons. The details are plotted in Fig. 4.

Note that a decaying system can be viewed as an open quantum process^45^, i.e. an interaction with an environment is modelled by particular operators resulting in a non-unitary evolution of the system of interest. This gives another perspective of the physics behind the decay.

### Entanglement properties of the mixed 3-photon-states

In case spin is not a proper quantum number, all three possible spin eigenstates ${s}_{\hat{\overrightarrow{n}}}=0,\,+1,\,-1$ are equally probable, then the resulting state is

20 $${\rho }_{mixed}(p,\,{{\rm{\Phi }}}_{plane})=p|{{\rm{\Psi }}}_{{s}_{\hat{\overrightarrow{n}}}=0}\rangle \langle {{\rm{\Psi }}}_{{s}_{\hat{\overrightarrow{n}}}=0}|+\frac{1-p}{2}|{{\rm{\Psi }}}_{{s}_{\hat{\overrightarrow{n}}}=+1}\rangle \langle {{\rm{\Psi }}}_{{s}_{\hat{\overrightarrow{n}}}=+1}|+\frac{1-p}{2}|{{\rm{\Psi }}}_{{s}_{\hat{\overrightarrow{n}}}=-1}\rangle \langle {{\rm{\Psi }}}_{{s}_{\hat{\overrightarrow{n}}}=-1}|$$

with $p=\frac{1}{3}$. Computing the mixedness *Trρ* ^2^ we find for $p=1,\,\frac{1}{3}$ no dependence on Φ_*plane*_. This is also the case for the entanglement properties of the state, thus without loss of generality we can set Φ_*plane*_ = 0.

**Figure 5:**
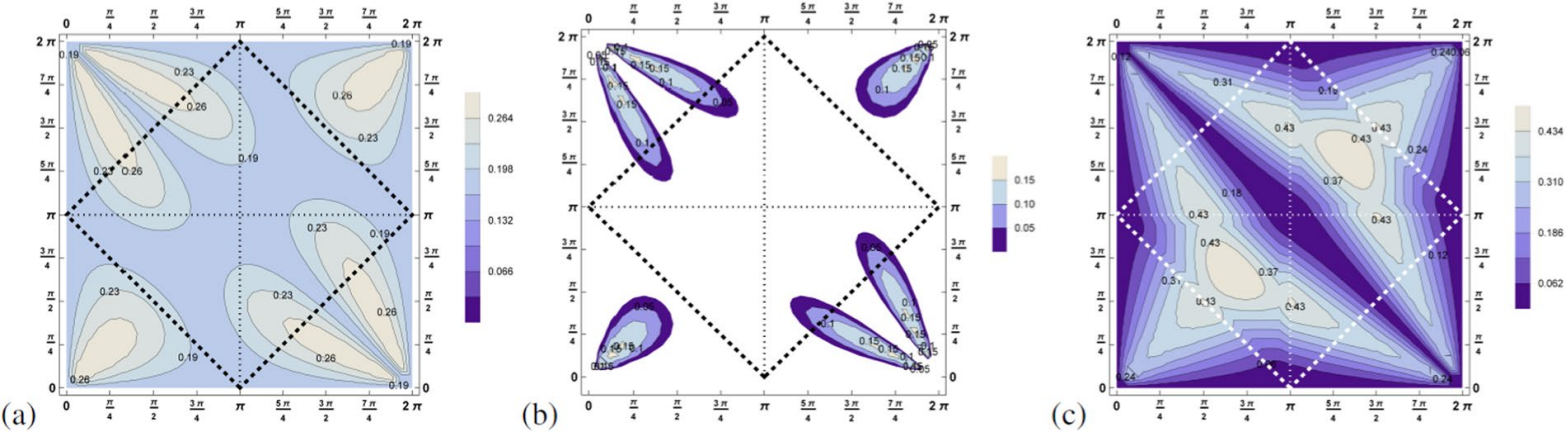
These three contour plots show (**a**) *Q*_*SEP*_, (**b**) *Q*_*GHZ*_ and (**c**) *Q*_*W*_ for the state mixed equally between all three possible quantum states ${s}_{\hat{\overrightarrow{n}}}=\mathrm{0,}\,+1,\,-1$, equation 17. Still genuine multipartite entanglement is revealed for some scenarios $({\tilde{{\rm{\Theta }}}}_{ab},\,{\tilde{{\rm{\Theta }}}}_{bc})$. The criterion *Q*_*W*_ detecting *W*-type of genuine multipartite entanglement is by far more sensitive to reveal genuine multipartite entanglement.

The three criteria *Q*_*SEP*_, *Q*_*GHZ*_ and *Q*_*W*_ are presented in Fig. 5. Remarkably, entanglement is not lost for any setup, however, genuine multipartite entanglement is more sensitive to the angles. Concerning the physical attainable region we find that *Q*_*SEP*_ attains in good approximation a constant value (*Q*_*SEP*_ = [0.17, 0.2]) which shows a kind of symmetrization in the sense that the difference between the relevant off diagonal element and the sum of the relevant diagonal elements of the density matrix is constant, see equation (14), however, it is strictly non-positive for *Q*_*GHZ*_. In strong contrast, *Q*_*W*_ reveals differences in the entanglement properties since it still varies strongly with the angles. Consequently, this criterion reveals refined properties of the system under investigation. This proves that entanglement properties of the state can be revealed in the highly mixed scenario and gives the hope that biological properties of the system may be obtainable.

In summary, *GHZ*-type entanglement can no longer be found in a physically available region, however, *W*-type entanglement is robust against this mixing. Particularly, in the fully symmetric case ${\tilde{{\rm{\Theta }}}}_{ab}={\tilde{{\rm{\Theta }}}}_{bc}=\frac{2\pi }{3}$ we find a local maximum with the value *Q*_*W*_ = 0.5. This shows that the dynamics of the decay process does not wash out fully the entanglement features and favours Dicke-type entanglement over GHZ-type entanglement. Thus the decay process favours a symmetrization among the three photons enabling bipartite entanglement. From the theoretical point of view different classes of multipartite entangled states cannot be converted into each other by local operations and classical communication (LOCC), only non-local operations could do the job. The mixing can thus be seen as a procedure resulting in a state where tracing out one photon does not lead to a separable state, i.e. transforming to a certain type of genuinely multipartite entangled state maximizing bipartite entanglement, i.e. a *W*-type state (defined as being detected by *Q*_*W*_ but not *Q*_*GHZ*_). The process is most symmetric for a spatial symmetric state (${\tilde{{\rm{\Theta }}}}_{ab}={\tilde{{\rm{\Theta }}}}_{bc}=\frac{2\pi }{3}$). Thus the spatial symmetry of the three momenta superposes the symmetry of the polarisation degrees of freedom which is obviously due to the close relationship between momenta and polarisation for relativistic massless particles (transversality condition).

## Discussion

Monitoring metabolic processes as well as distinct physical reactions related to chemical processes are key ingredients for exploring nature and its very workings. We analyze the entanglement properties in the polarisation degrees of freedom of three photons resulting from the decay of ortho–positronium in the full parameter space. For this we use the HMGH-framework which allows for entanglement detection in addition to refinements such as *W*-type or more generally *Dicke*-type entanglement versus *GHZ*-type entanglement. The framework provides non-linear entanglement witnesses based on local observables, i.e. it does not need full information on the state that in many cases is not attainable.

In particular, for a definite spin value of ortho-positronium we find that entanglement and even stronger genuine multipartite entanglement is present for the full parameter space. Surprisingly, in the mixed scenario entanglement as well as genuine multipartite entanglement are not lost, however, only *W*-type genuine multipartite entanglement is detectable. This is due to the interplay of the kinematics of the three-body particle decay and the Bose-symmetry constraining the entanglement properties to favor *W*-type over *GHZ*-type genuine multipartite entanglement. Furthermore, whereas the criterion detecting entanglement, *Q*_*SEP*_, is in good approximation constant over the physical relevant region, this is not the case for the specific criterion *Q*_*W*_, which reveals different properties in dependence of the decay angles due to a specific “*symmetrisation process*” as an effect of the decay process. Since the mixing does not destroy entanglement per se and genuine multipartite entanglement is shown to be still dependent on the angles, this proves that entanglement can be related to physical processes and gives the hope that entanglement will be related to real biological processes.

J-PET, a Positron-Emission-Tomograph, relies on new technology enabling three-photon tomography^17–19^. This is due to a new detector scheme based on plastic scintillators^14^, novel digital sampling electronics^15,16^ and a development of trilateration-based reconstruction^19^. Consequently, J-PET gives a possibility to determine the linear polarization of high energy photons via the registration of the direction of the photon before and after its Compton scattering^16^. It allows for the measuring of correlations of photons with superior time and angular resolutions via Compton scattering. It is beyond the scope of this contribution to compute how the entanglement of the three photons can be revealed by the three Compton scattered photons, but we will tackle this problem in future work.
